# 3-Phenyl-1-(*p*-tol­yl)-1*H*-benzo[*f*]chromene benzene hemisolvate

**DOI:** 10.1107/S1600536811001668

**Published:** 2011-06-18

**Authors:** Meng Wei Xue

**Affiliations:** a Biochemical and Environmental Engineering College, Nanjing Xiaozhuang University, Nanjing 210017, People’s Republic of China

## Abstract

The title compound, C_26_H_20_O·0.5C_6_H_6_, was obtained from condensation reaction of 2-naphthol, 4-methyl­benzaldehyde and phenyl­methanamine. The naphthyl ring system is orented at dihedral angles of 84.11 (1) and 19.33 (8)° with respect to the mean planes of the two benzene rings.

## Related literature

For applications of Betti-type reactions, see: Wang *et al.* (2005 ▶). The reaction of substituted phenols and aldehydes under controlled conditions has been used to build up a compound with two chiral centers, see: Gardiner & Raston (1997 ▶); Gutsche & Nam (1998 ▶). For bond-length data, see: Allen *et al.* (1987 ▶).
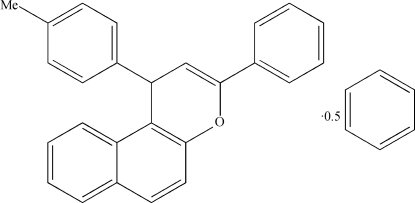


## Experimental

### 

#### Crystal data

C_26_H_20_O·0.5C_6_H_6_
*M* *_r_* = 387.47
Monoclinic, 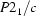

*a* = 12.653 (3) Å
*b* = 5.9049 (12) Å
*c* = 29.974 (8) Å
β = 109.08 (3)°
*V* = 2116.5 (9) Å^3^
*Z* = 4
Mo *K*α radiation
μ = 0.07 mm^−1^
*T* = 293 K
0.20 × 0.20 × 0.20 mm

#### Data collection

Rigaku Mercury2 diffractometer
Absorption correction: multi-scan (*CrystalClear*; Rigaku, 2005 ▶) *T*_min_ = 0.813, *T*_max_ = 1.000
14405 measured reflections
3623 independent reflections
1438 reflections with *I* > 2σ(*I*)
*R*_int_ = 0.148

#### Refinement

*R*[*F* ^2^ > 2σ(*F* ^2^)] = 0.080
*wR*(*F* ^2^) = 0.144
*S* = 0.93
3623 reflections
273 parameters
H-atom parameters constrained
Δρ_max_ = 0.18 e Å^−3^
Δρ_min_ = −0.20 e Å^−3^

### 

Data collection: *CrystalClear* (Rigaku, 2005 ▶); cell refinement: *CrystalClear*; data reduction: *CrystalClear*; program(s) used to solve structure: *SHELXS97* (Sheldrick, 2008 ▶); program(s) used to refine structure: *SHELXL97* (Sheldrick, 2008 ▶); molecular graphics: *SHELXTL* (Sheldrick, 2008 ▶); software used to prepare material for publication: *SHELXTL*.

## Supplementary Material

Crystal structure: contains datablock(s) I, global. DOI: 10.1107/S1600536811001668/jh2252sup1.cif

Structure factors: contains datablock(s) I. DOI: 10.1107/S1600536811001668/jh2252Isup2.hkl

Supplementary material file. DOI: 10.1107/S1600536811001668/jh2252Isup3.cml

Additional supplementary materials:  crystallographic information; 3D view; checkCIF report

## Figures and Tables

**Fig. 1.**
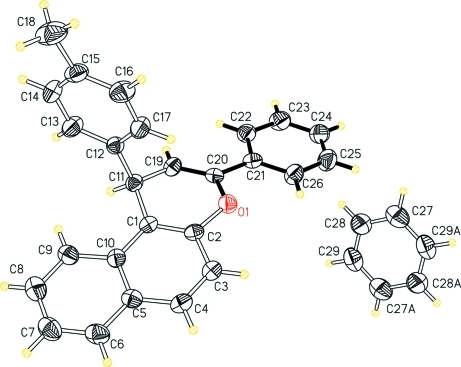
Perspective structure of the title compound, showing the atom-numbering scheme. Displacement ellipsoids are drawn at the 30% probability level.

## supplementary crystallographic information

### Comment

The reaction of substituted phenols and aldehydes under controlled conditions has been used to build up supramolecular compounds, the most important ones being calixarenes (Gardiner & Raston *et al.* 1997, Gutsche & Nam *et al.* 1998). 2-Naphthol reacts with aromatic aldehydes to produce 14-aryl-14*H*-dibenzo[a,j]xanthenes, which could be used as anti-inflamatory agents. Here we report the synthesis and crystal structure of the title compound. The asymmetric unit of the compound contains a benzene solvent molecule (Fig. 1). The bond lengths and angles are within normal ranges (Allen *et al.* 1987).

Rings of the two benzenes and naphthol are, of course, planar. The dihedral angles between rings A (C1–C10) and B (C12–C17), and between rings A and C (C21–C26), are 84.11 (1) and 19.33 (8), respectively. The orientation of ring B with respect to the mean planes of the two groups containing rings A and C, may be described by the dihedral angles of 84.11 (1) and 76.82 (2), respectively. The molecules are stabilized by intramolecular C—H···O hydrogen bonding (Table 1). Intermolecular attractions are only on the order of Van der Waals forces.

### Experimental

4-Methylbenzaldehyde (1.8 g, 0.015 mol) and phenylmethanamine (1.605 g, 0.015 mol) was added to 2-naphthol (2.16 g, 0.015 mol) without solvent under nitrogen. The temperature was raised to 120°C in one hour gradually and the mixture was stirred at this temperature for 12 h. The system was treated with 30 ml of ethanol 95% and cooled. The precipitate was filtered and washed with a small amount of ethanol 95%. The title compound was isolated using column chromatography (Petroleum ether: ethyl acetate-4:1). Single crystals suitable for X-ray diffraction analysis were obtained from slow evaporation of a solution of the title compound in b enzeneat room temperature.

### Refinement

H atoms bonded to O atoms were located in a difference map and refined freely. Other H atoms were positioned geometrically and refined using a riding model, with C—H = 0.93–0.96 Å and *U*_iso_(H) = 1.2*U*_eq_(C).

### Crystal data

**Table:**

C_26_H_20_O·0.5C_6_H_6_	*F*(000) = 820
*M**_r_* = 387.47	*D*_x_ = 1.216 Mg m^−^^3^
Monoclinic, *P*2_1_/*c*	Mo *K*α radiation, λ = 0.71073 Å
Hall symbol: -P 2ybc	Cell parameters from 3623 reflections
*a* = 12.653 (3) Å	θ = 2.6–24.8°
*b* = 5.9049 (12) Å	µ = 0.07 mm^−^^1^
*c* = 29.974 (8) Å	*T* = 293 K
β = 109.08 (3)°	Prism, colorless
*V* = 2116.5 (9) Å^3^	0.20 × 0.20 × 0.20 mm
*Z* = 4	

### Data collection

**Table:**

Rigaku Mercury2 diffractometer	3623 independent reflections
Radiation source: fine-focus sealed tube	1438 reflections with *I* > 2σ(*I*)
graphite	*R*_int_ = 0.148
Detector resolution: 13.6612 pixels mm^-1^	θ_max_ = 24.8°, θ_min_ = 3.2°
CCD_Profile_fitting scans	*h* = −14→13
Absorption correction: multi-scan (*CrystalClear*; Rigaku, 2005)	*k* = −6→6
*T*_min_ = 0.813, *T*_max_ = 1.000	*l* = −35→35
14405 measured reflections	

### Refinement

**Table:**

Refinement on *F*^2^	Secondary atom site location: difference Fourier map
Least-squares matrix: full	Hydrogen site location: inferred from neighbouring sites
*R*[*F*^2^ > 2σ(*F*^2^)] = 0.080	H-atom parameters constrained
*wR*(*F*^2^) = 0.144	*w* = 1/[σ^2^(*F*_o_^2^) + (0.010*P*)^2^ + 1.9*P*] where *P* = (*F*_o_^2^ + 2*F*_c_^2^)/3
*S* = 0.93	(Δ/σ)_max_ < 0.001
3623 reflections	Δρ_max_ = 0.18 e Å^−^^3^
273 parameters	Δρ_min_ = −0.20 e Å^−^^3^
0 restraints	Extinction correction: *SHELXL97* (Sheldrick, 2008), Fc^*^=kFc[1+0.001xFc^2^λ^3^/sin(2θ)]^-1/4^
Primary atom site location: structure-invariant direct methods	Extinction coefficient: 0.0054 (6)

### Special details

**Table:**

Geometry. All e.s.d.'s (except the e.s.d. in the dihedral angle between two l.s. planes) are estimated using the full covariance matrix. The cell e.s.d.'s are taken into account individually in the estimation of e.s.d.'s in distances, angles and torsion angles; correlations between e.s.d.'s in cell parameters are only used when they are defined by crystal symmetry. An approximate (isotropic) treatment of cell e.s.d.'s is used for estimating e.s.d.'s involving l.s. planes.
Refinement. Refinement of *F*^2^ against ALL reflections. The weighted *R*-factor *wR* and goodness of fit *S* are based on *F*^2^, conventional *R*-factors *R* are based on *F*, with *F* set to zero for negative *F*^2^. The threshold expression of *F*^2^ > σ(*F*^2^) is used only for calculating *R*-factors(gt) *etc*. and is not relevant to the choice of reflections for refinement. *R*-factors based on *F*^2^ are statistically about twice as large as those based on *F*, and *R*- factors based on ALL data will be even larger.

### Fractional atomic coordinates and isotropic or equivalent isotropic displacement parameters (Å^2^)

**Table:**

	*x*	*y*	*z*	*U*_iso_*/*U*_eq_	
O1	0.1992 (2)	1.0645 (5)	0.23039 (9)	0.0549 (8)	
C5	−0.1441 (4)	1.0682 (8)	0.16184 (15)	0.0502 (12)	
C10	−0.0792 (4)	0.8897 (8)	0.15186 (14)	0.0464 (12)	
C1	0.0391 (4)	0.8852 (7)	0.17364 (14)	0.0433 (11)	
C11	0.1128 (3)	0.7117 (7)	0.16231 (13)	0.0464 (12)	
H11A	0.0762	0.5639	0.1601	0.056*	
C12	0.1302 (3)	0.7595 (8)	0.11524 (14)	0.0452 (11)	
C2	0.0853 (4)	1.0539 (8)	0.20516 (14)	0.0468 (12)	
C9	−0.1366 (4)	0.7168 (8)	0.12064 (15)	0.0591 (13)	
H9A	−0.0957	0.5997	0.1135	0.071*	
C3	0.0233 (4)	1.2317 (7)	0.21550 (14)	0.0497 (12)	
H3A	0.0589	1.3428	0.2372	0.060*	
C6	−0.2621 (4)	1.0666 (9)	0.13957 (16)	0.0664 (15)	
H6A	−0.3047	1.1837	0.1455	0.080*	
C4	−0.0887 (4)	1.2401 (8)	0.19354 (14)	0.0557 (13)	
H4A	−0.1296	1.3606	0.1994	0.067*	
C8	−0.2511 (5)	0.7169 (9)	0.10062 (16)	0.0689 (15)	
H8A	−0.2870	0.5981	0.0811	0.083*	
C13	0.1067 (4)	0.6008 (8)	0.08004 (16)	0.0673 (15)	
H13A	0.0820	0.4582	0.0854	0.081*	
C17	0.1694 (4)	0.9652 (8)	0.10589 (16)	0.0619 (14)	
H17A	0.1881	1.0752	0.1294	0.074*	
C15	0.1555 (4)	0.8569 (10)	0.02733 (16)	0.0649 (15)	
C7	−0.3138 (4)	0.8968 (10)	0.10966 (17)	0.0737 (16)	
H7A	−0.3910	0.9000	0.0952	0.088*	
C14	0.1188 (4)	0.6477 (10)	0.03650 (17)	0.0752 (16)	
H14A	0.1020	0.5365	0.0133	0.090*	
C18	0.1597 (4)	0.9155 (10)	−0.02193 (15)	0.103 (2)	
H18A	0.1856	1.0682	−0.0220	0.155*	
H18B	0.0862	0.9012	−0.0446	0.155*	
H18C	0.2098	0.8137	−0.0300	0.155*	
C16	0.1819 (4)	1.0139 (9)	0.06301 (18)	0.0712 (16)	
H16A	0.2086	1.1554	0.0582	0.085*	
C27	0.5726 (5)	1.0641 (15)	0.4766 (2)	0.097 (2)	
H27A	0.6217	1.1072	0.4609	0.117*	
C28	0.5207 (6)	0.8584 (14)	0.4683 (2)	0.096 (2)	
H28A	0.5347	0.7606	0.4466	0.115*	
C29	0.4494 (5)	0.7940 (10)	0.4912 (3)	0.0953 (19)	
H29A	0.4151	0.6528	0.4850	0.114*	
C25	0.4963 (4)	1.0693 (9)	0.33788 (16)	0.0753 (16)	
H25A	0.5136	1.1949	0.3577	0.090*	
C26	0.3953 (4)	1.0623 (9)	0.30149 (15)	0.0639 (14)	
H26A	0.3452	1.1819	0.2973	0.077*	
C21	0.3688 (4)	0.8798 (8)	0.27155 (15)	0.0491 (12)	
C23	0.5458 (4)	0.7128 (9)	0.31572 (17)	0.0740 (16)	
H23A	0.5962	0.5933	0.3204	0.089*	
C24	0.5710 (4)	0.8955 (10)	0.34533 (16)	0.0727 (16)	
H24A	0.6381	0.9011	0.3702	0.087*	
C22	0.4449 (4)	0.7045 (8)	0.27852 (15)	0.0615 (14)	
H22A	0.4288	0.5804	0.2583	0.074*	
C20	0.2610 (4)	0.8690 (8)	0.23220 (14)	0.0444 (11)	
C19	0.2214 (4)	0.7014 (8)	0.20194 (14)	0.0503 (12)	
H19A	0.2636	0.5696	0.2056	0.060*	

### Atomic displacement parameters (Å^2^)

**Table:**

	*U*^11^	*U*^22^	*U*^33^	*U*^12^	*U*^13^	*U*^23^
O1	0.045 (2)	0.052 (2)	0.063 (2)	0.0056 (18)	0.0123 (16)	−0.0046 (16)
C5	0.052 (4)	0.057 (4)	0.044 (3)	0.002 (3)	0.019 (3)	0.012 (2)
C10	0.054 (3)	0.050 (3)	0.037 (3)	−0.004 (3)	0.017 (2)	0.006 (2)
C1	0.051 (3)	0.041 (3)	0.041 (3)	0.005 (2)	0.020 (2)	0.002 (2)
C11	0.054 (3)	0.045 (3)	0.042 (3)	−0.003 (2)	0.019 (2)	−0.001 (2)
C12	0.045 (3)	0.046 (3)	0.045 (3)	0.002 (2)	0.015 (2)	−0.002 (2)
C2	0.051 (3)	0.050 (3)	0.044 (3)	0.005 (3)	0.023 (3)	0.004 (2)
C9	0.051 (4)	0.072 (4)	0.053 (3)	0.000 (3)	0.016 (3)	0.009 (3)
C3	0.058 (3)	0.047 (3)	0.045 (3)	0.005 (3)	0.017 (2)	0.004 (2)
C6	0.053 (4)	0.087 (4)	0.058 (3)	0.017 (3)	0.016 (3)	0.015 (3)
C4	0.064 (4)	0.058 (4)	0.048 (3)	0.013 (3)	0.023 (3)	0.003 (3)
C8	0.069 (4)	0.071 (4)	0.061 (3)	−0.008 (3)	0.013 (3)	0.010 (3)
C13	0.087 (4)	0.060 (4)	0.063 (3)	−0.004 (3)	0.036 (3)	−0.010 (3)
C17	0.081 (4)	0.055 (4)	0.054 (3)	−0.011 (3)	0.028 (3)	−0.006 (3)
C15	0.059 (4)	0.093 (5)	0.047 (3)	0.013 (3)	0.024 (3)	0.005 (3)
C7	0.057 (4)	0.094 (5)	0.065 (4)	−0.003 (4)	0.013 (3)	0.015 (3)
C14	0.081 (4)	0.092 (5)	0.053 (3)	−0.009 (3)	0.022 (3)	−0.024 (3)
C18	0.101 (5)	0.157 (6)	0.061 (4)	0.027 (4)	0.040 (3)	0.025 (4)
C16	0.088 (4)	0.068 (4)	0.069 (4)	0.002 (3)	0.041 (3)	0.011 (3)
C27	0.082 (5)	0.116 (6)	0.093 (5)	−0.011 (5)	0.027 (4)	0.016 (4)
C28	0.080 (5)	0.112 (7)	0.093 (5)	−0.003 (4)	0.026 (4)	−0.014 (4)
C29	0.082 (5)	0.082 (5)	0.111 (6)	−0.018 (4)	0.017 (4)	−0.002 (4)
C25	0.075 (4)	0.085 (5)	0.059 (4)	0.001 (4)	0.012 (3)	−0.012 (3)
C26	0.060 (4)	0.070 (4)	0.053 (3)	0.014 (3)	0.007 (3)	−0.006 (3)
C21	0.049 (3)	0.056 (3)	0.047 (3)	0.009 (3)	0.023 (2)	0.007 (3)
C23	0.059 (4)	0.095 (5)	0.063 (4)	0.020 (3)	0.012 (3)	0.003 (3)
C24	0.054 (4)	0.110 (5)	0.047 (3)	0.003 (4)	0.007 (3)	−0.004 (3)
C22	0.054 (4)	0.070 (4)	0.058 (3)	0.008 (3)	0.015 (3)	−0.003 (3)
C20	0.039 (3)	0.051 (3)	0.048 (3)	0.005 (3)	0.019 (2)	0.006 (2)
C19	0.058 (3)	0.048 (3)	0.042 (3)	0.007 (3)	0.013 (2)	0.002 (2)

### Geometric parameters (Å, °)

**Table:**

O1—C20	1.386 (4)	C15—C14	1.379 (6)
O1—C2	1.392 (5)	C15—C18	1.534 (6)
C5—C4	1.410 (5)	C7—H7A	0.9300
C5—C6	1.422 (6)	C14—H14A	0.9300
C5—C10	1.427 (5)	C18—H18A	0.9600
C10—C9	1.415 (5)	C18—H18B	0.9600
C10—C1	1.424 (5)	C18—H18C	0.9600
C1—C2	1.366 (5)	C16—H16A	0.9300
C1—C11	1.498 (5)	C27—C28	1.364 (7)
C11—C19	1.495 (5)	C27—C29^i^	1.374 (7)
C11—C12	1.523 (5)	C27—H27A	0.9300
C11—H11A	0.9800	C28—C29	1.353 (7)
C12—C13	1.369 (5)	C28—H28A	0.9300
C12—C17	1.375 (5)	C29—C27^i^	1.374 (7)
C2—C3	1.405 (5)	C29—H29A	0.9300
C9—C8	1.376 (6)	C25—C24	1.363 (6)
C9—H9A	0.9300	C25—C26	1.383 (5)
C3—C4	1.355 (5)	C25—H25A	0.9300
C3—H3A	0.9300	C26—C21	1.372 (5)
C6—C7	1.361 (6)	C26—H26A	0.9300
C6—H6A	0.9300	C21—C22	1.383 (5)
C4—H4A	0.9300	C21—C20	1.484 (5)
C8—C7	1.404 (6)	C23—C24	1.367 (6)
C8—H8A	0.9300	C23—C22	1.394 (5)
C13—C14	1.391 (6)	C23—H23A	0.9300
C13—H13A	0.9300	C24—H24A	0.9300
C17—C16	1.376 (5)	C22—H22A	0.9300
C17—H17A	0.9300	C20—C19	1.325 (5)
C15—C16	1.372 (6)	C19—H19A	0.9300
			
C20—O1—C2	117.0 (3)	C6—C7—H7A	119.9
C4—C5—C6	122.4 (5)	C8—C7—H7A	119.9
C4—C5—C10	118.6 (4)	C15—C14—C13	120.9 (5)
C6—C5—C10	119.0 (5)	C15—C14—H14A	119.6
C9—C10—C1	121.6 (4)	C13—C14—H14A	119.6
C9—C10—C5	117.8 (4)	C15—C18—H18A	109.5
C1—C10—C5	120.6 (4)	C15—C18—H18B	109.5
C2—C1—C10	116.9 (4)	H18A—C18—H18B	109.5
C2—C1—C11	119.8 (4)	C15—C18—H18C	109.5
C10—C1—C11	123.3 (4)	H18A—C18—H18C	109.5
C19—C11—C1	109.1 (3)	H18B—C18—H18C	109.5
C19—C11—C12	111.7 (3)	C15—C16—C17	120.9 (5)
C1—C11—C12	111.9 (3)	C15—C16—H16A	119.5
C19—C11—H11A	108.0	C17—C16—H16A	119.5
C1—C11—H11A	108.0	C28—C27—C29^i^	118.3 (6)
C12—C11—H11A	108.0	C28—C27—H27A	120.8
C13—C12—C17	116.7 (4)	C29^i^—C27—H27A	120.8
C13—C12—C11	121.7 (4)	C29—C28—C27	121.3 (6)
C17—C12—C11	121.6 (4)	C29—C28—H28A	119.4
C1—C2—O1	122.8 (4)	C27—C28—H28A	119.4
C1—C2—C3	123.6 (4)	C28—C29—C27^i^	120.4 (6)
O1—C2—C3	113.6 (4)	C28—C29—H29A	119.8
C8—C9—C10	121.9 (5)	C27^i^—C29—H29A	119.8
C8—C9—H9A	119.1	C24—C25—C26	121.3 (5)
C10—C9—H9A	119.1	C24—C25—H25A	119.4
C4—C3—C2	119.4 (4)	C26—C25—H25A	119.4
C4—C3—H3A	120.3	C21—C26—C25	120.3 (5)
C2—C3—H3A	120.3	C21—C26—H26A	119.8
C7—C6—C5	121.3 (5)	C25—C26—H26A	119.8
C7—C6—H6A	119.4	C26—C21—C22	118.7 (4)
C5—C6—H6A	119.4	C26—C21—C20	121.2 (4)
C3—C4—C5	120.8 (4)	C22—C21—C20	120.1 (4)
C3—C4—H4A	119.6	C24—C23—C22	120.3 (5)
C5—C4—H4A	119.6	C24—C23—H23A	119.8
C9—C8—C7	119.8 (5)	C22—C23—H23A	119.8
C9—C8—H8A	120.1	C25—C24—C23	119.1 (5)
C7—C8—H8A	120.1	C25—C24—H24A	120.5
C12—C13—C14	121.6 (5)	C23—C24—H24A	120.5
C12—C13—H13A	119.2	C21—C22—C23	120.3 (5)
C14—C13—H13A	119.2	C21—C22—H22A	119.8
C12—C17—C16	122.3 (4)	C23—C22—H22A	119.8
C12—C17—H17A	118.8	C19—C20—O1	120.9 (4)
C16—C17—H17A	118.8	C19—C20—C21	128.3 (4)
C16—C15—C14	117.5 (4)	O1—C20—C21	110.8 (4)
C16—C15—C18	121.5 (5)	C20—C19—C11	123.8 (4)
C14—C15—C18	120.9 (5)	C20—C19—H19A	118.1
C6—C7—C8	120.3 (5)	C11—C19—H19A	118.1

Symmetry codes: (i) −*x*+1, −*y*+2, −*z*+1.

### Hydrogen-bond geometry (Å, °)

**Table:**

*D*—H···*A*	*D*—H	H···*A*	*D*···*A*	*D*—H···*A*
C26—H26A···O1	0.93	2.34	2.692 (6)	102.
